# Understanding learners’ metacognitive experiences in learning to write in English as a foreign language: A structural equation modeling approach

**DOI:** 10.3389/fpsyg.2022.986301

**Published:** 2022-09-02

**Authors:** Qiyu Sun, Lawrence Jun Zhang

**Affiliations:** ^1^School of Foreign Language Education, Jilin University, Changchun, China; ^2^Faculty of Education and Social Work, The University of Auckland, Auckland, New Zealand

**Keywords:** ESL/EFL writing, metacognition, English as a foreign/second language, questionnaire development, metacognitive experiences

## Abstract

Many researchers have acknowledged the role of metacognition in facilitating learning to write in English as a foreign language (EFL). Although research on metacognition has explored learners’ metacognitive knowledge and metacognitive strategies in the field of EFL writing, little is known about the nature of learners’ metacognitive experiences in EFL writing. To fill such an important gap, this study was designed to assess EFL learners’ metacognitive experiences before, during, and after writing. Data were collected from a total of 760 undergraduates through three self-report questionnaires and a writing task. Results from quantitative analyses showed four subcategories of EFL learners’ metacognitive experiences in writing: metacognitive feeling, metacognitive judgments/estimates, online task-specific metacognitive knowledge, and online task-specific metacognitive strategies. Based on the empirical evidence, we propose a model of metacognitive experiences in EFL writing. Theoretical, methodological, and pedagogical implications are discussed.

## Introduction

Writing, as a self-planned and self-sustained process, requires a high degree of accuracy and logic involving not only the use of vocabulary and grammar but also skills in organizing thoughts and ideas (Zimmerman and Risemberg, 1997; Teng and Zhang, 2016, 2020; Sun and Wang, 2020; Zhang and Cheng, 2021). The complex process of writing involves cognitive, metacognitive, affective, and behavioral maneuvers (Teng and Zhang, 2020; Sun et al., 2021; Chen et al., 2022; Sun and Zhang, 2022; Teng et al., 2022). Researchers have investigated these maneuvers, of which metacognition is crucial for language learning success, to develop learners’ writing proficiency (Zhang, 2010; Zhang and Zhang, 2019; Alfaifi, 2021; Zhang et al., 2021a).

Metacognition, as an integral component of self-regulated learning, enables learners to monitor and control their cognitive processes, and develop self-awareness of the learning process (Zhang and Zhang, 2019; Sun and Zhang, 2022; Teng et al., 2022). Metacognition comprising metacognitive knowledge, metacognitive experiences, and metacognitive strategies, has been widely recognized to have a bearing on the second language (L2) learning and development (Qin and Zhang, 2019; Zhang et al., 2019; Teng and Zhang, 2020; Wu, 2021; Zhang and Zhang, 2022). Wenden’s (1987) early seminal work on metacognition provided insight into L2 teaching and learning, attracting much attention from L2 writing researchers and instructors in their debate and discussion on the theoretical (e.g., Lee and Mak, 2018; Zhang and Zhang, 2018, 2019) and practical implications (e.g., Negretti and McGrath, 2018; Sun et al., 2021; Zhang and Zhang, 2022). While much of the research on metacognition in L2 writing has focused on the nature and the role of metacognitive knowledge (e.g., Ruan, 2014; Teng, 2020; Teng and Zhang, 2021) and metacognitive strategies (e.g., Qin and Zhang, 2019; Zhao and Liao, 2021; Zhang and Zhang, 2022), there are still issues that need to be addressed.

Despite the facilitating role of metacognitive knowledge and metacognitive strategies in L2 writing development, as noted above, little attention has been paid to the nature of metacognitive experiences in learning to write, particularly for English as a foreign language (EFL) learners who are exposed to English in the classroom setting. This study, situated in an EFL learning context, attempts to fill the research gaps by assessing EFL learners’ metacognitive experiences before, during, and after the writing process using a structural equation modeling approach. The findings of this study are expected to map out EFL learners’ metacognitive experiences in writing as well as provide pedagogical implications for EFL writing instruction.

## Literature review

### Metacognition and L2 writing

Metacognition refers to individuals’ self-awareness of their own cognitive processes and their ability to organize these processes (Flavell, 1976, 1979; Teng et al., 2021, 2022; Wu, 2021; Zhang et al., 2021b). According to Papaleontiou-Louca (2003), metacognition depicts not only individuals’ awareness and control of cognitive processes but also their emotions and motivations (see also Efklides, 2017; Efklides et al., 2017; Sun and Zhang, 2022). Researchers have unanimously accepted that metacognition could monitor and control cognitive processes, reflecting learners’ abilities in self-regulated learning (Flavell, 1979; Tarricone, 2011; Zhang and Zhang, 2019; Teng et al., 2021).

Metacognition incorporates three essential components: metacognitive knowledge, metacognitive strategies, and metacognitive experiences (Flavell, 1979; Zhang, 2010; Teng, 2020; Wu, 2021; Sun and Zhang, 2022; Teng and Zhang, 2022). Metacognitive knowledge is a type of knowledge retrieved from long-term memory for learners to know about themselves and other cognitive processors as well as their relationships with cognitive tasks, goals, actions, and experiences (Flavell, 1979; Efklides, 2001). Metacognitive knowledge is a fundamental component of metacognition and a prerequisite for self-regulated learning, consisting of *person knowledge, task knowledge, and strategy knowledge* (Flavell, 1979; Zhang and Zhang, 2019). Metacognitive strategies refer to the skills used when individuals deliberately monitor their own cognitive progress related to learning or fulfilling tasks. Metacognitive strategies comprise *planning, monitoring, and evaluating* during the learning process (Brown, 1978; Flavell, 1979; Schraw, 1998; Lee and Mak, 2018). To enhance the language learning process, learners make use of metacognitive strategies for “overseeing, regulating, and directing the language learning task, and thinking about the process of learning” (Zhang, 2010, p. 321).

In addition to metacognitive knowledge and strategies, metacognitive experiences are what the individual goes through during a cognitive endeavor, including cognitive and affective experiences that are online metacognitive knowledge, ideas and beliefs, feelings, goals, and self-judgments (Flavell, 1979; Efklides, 2001; Tarricone, 2011). Metacognitive experiences, the focus of our study, involve learners’ current and ongoing/online cognition and their emotions (Efklides, 2006a, 2017; Sun et al., 2021; Sun and Zhang, 2022). In our study, *emotions* are synonymous with *feelings*. Metacognitive experiences related to individuals’ working memory can happen before, during, and after the cognitive process, involving prospective and retrospective perspectives (Efklides, 2002a,b, 2009). Specifically, metacognitive experiences, on the one hand, are the subjective experiences of thinking ahead to a cognitive task; on the other hand, metacognitive experiences occur during and after the cognitive task about what the cognition involved (Efklides, 2002b; Efklides and Vlachopoulos, 2012).

In the field of educational psychology, Efklides (2002a,b) developed a framework of metacognitive experiences comprising three subcategories, namely, *metacognitive feelings, metacognitive judgments/estimates, and online task-specific knowledge*. Metacognitive feelings have been classified into the *feeling of difficulty* that occurs when a task seems too difficult (Efklides, 2002a); the *feeling of familiarity* regarding the previous occurrence of a stimulus and fluency of processing (Nelson, 1996); the *feeling of confidence* denoting the feeling that individuals trust themselves; the *feeling of satisfaction* when an outcome meets the criteria regarding its quality of the answer (Efklides, 2002a); and the *feeling of knowing* relating to a tip-of-tongue phenomenon. Metacognitive judgments/estimates include (1) *judgment of learning*; (2) *estimate of solution correctness*; (3) *estimate of time expenditure*; (4) *estimate of effort expenditure*; and (5) *episodic memory judgment* (Efklides, 2001, 2006a, 2009). Judgment of learning emphasizes the judgment made by a learner in the learning process. An estimate of solution correctness focuses on the quality of answers; an estimate of time expenditure denotes the time learners expect to spend on completing tasks; an estimate of effort expenditure refers to learners’ effort allocation. An episodic memory judgment refers to learners’ evaluation of their memory-sourced information. Online task-specific knowledge relates to the awareness in real time of task characteristics and task-related knowledge about tasks and strategies (Efklides, 2001, 2002a, 2006a).

Taken together, metacognitive experiences, metacognitive knowledge, and metacognitive strategies have interactive relationships with each other, and there is no definite demarcation between them (Flavell, 1979; Anderson, 2002; Efklides, 2006a; Teng, 2020). Understanding the intricate relationship among the three components of metacognition is helpful for enhancing learners’ learning performance (Tarricone, 2011; Zhang and Zhang, 2019; Wu, 2021). Specifically, in the process of L2 learning, metacognitive knowledge affects learners’ metacognitive experiences and promotes the use of cognitive and metacognitive strategies (Garner, 1994; Lee and Mak, 2018; Zhang and Zhang, 2018, 2019); metacognitive experiences instigate the modification of metacognitive knowledge and the application of metacognitive strategies. In addition, metacognitive strategies accumulate to enrich learners’ metacognitive knowledge and thus influence their metacognitive experiences.

Given the important role of metacognition in L2 learning, researchers have identified the intertwined relationship between metacognition and writing. Writing is a complex problem-solving process in which writers conduct mental operations to achieve writing goals (Harris et al., 2009). Understanding the relationship between metacognition and writing provides insights into L2 writing teaching and learning, with a substantial body of empirical research indicating that developing L2 learners’ metacognitive competence can improve their writing quality (Yeh, 2015; Negretti and McGrath, 2018; Sun et al., 2021; Sun and Zhang, 2022; Teng et al., 2022). Previous studies have mainly established the basis for understanding the role of metacognitive knowledge (e.g., Negretti and McGrath, 2018; Teng, 2020) and metacognitive strategies (e.g., Zhang and Qin, 2018; Zhang and Zhang, 2022) in L2 writing. Kasper (1997), for instance, conducted an in-depth study where participants wrote short autobiographies and completed cognitive style questionnaires to investigate the role of metacognitive knowledge in L2 writing. Results revealed that learners’ strategy knowledge was related to their writing performance. Recently, Shih and Huang (2020) investigated metacognitive strategies in a flipped classroom setting. They explored EFL writers’ underlying factors of metacognitive strategy use in a university flipped classroom through writing accounts, classroom observations, and semi-structured interviews. Results indicated that students had five major metacognitive strategies: planning, self-monitoring, self-evaluation, directed attention, and selective attention. In addition, students’ expected learning outcomes and peer learning affected their metacognitive strategy use.

The above studies have supported the facilitative role of metacognitive knowledge and metacognitive strategies in L2 writing. Some prior studies, however, have produced mixed results on the L2 writing process as these studies were conducted in different learning contexts (Karlen, 2017; Zhao and Liao, 2021; Teng et al., 2022). In addition, metacognitive knowledge and metacognitive strategies have not been sufficiently discussed, specifically in the context of EFL writing, arguably, one of the most challenging learning settings.

### Metacognitive experiences and academic achievement

Our study attempts to contribute to the understanding of the nature of metacognitive experiences in writing. We reviewed prior studies on metacognitive experiences to understand the effects of metacognitive experiences on academic achievement in subject-specific teaching and learning, for instance, mathematics and L2 learning.

Efklides (2002a,b) began the investigation by developing a semantic scale exclusively measuring prospective and retrospective metacognitive experiences in mathematics. She proposed that metacognitive experiences incorporated metacognitive feelings, metacognitive judgments/estimates, and online task-specific metacognitive knowledge. Efklides and her associates also conducted a series of studies to investigate metacognitive experiences (e.g., Efklides and Vauras, 1999; Efklides and Misailidi, 2010; Efklides et al., 2017), which led to a deeper and broader understanding of metacognitive experiences in the general learning process. Following on from Efklides’ (2002a,b) seminal work, other researchers have strenuously explored metacognitive experiences in mathematics (e.g., Akama and Yamauchi, 2004; Akama, 2007). Recently, Aşık and Erktin (2019), using Efklides’ (2002a,b) questionnaire, investigated the mediating role of metacognitive experiences in the relationship between metacognitive knowledge and mathematical problem-solving. Their research generated six factors: problem-solving performance, feeling of understanding, feeling of familiarity, feeling of difficulty, estimated effort, and predicted solution correctness. They also found students’ metacognitive experiences affected mediating metacognitive experiences and task performance.

Despite a range of empirical studies on metacognitive experiences in general teaching and learning, research into metacognitive experiences in L2 learning is rather limited. One study on metacognitive experiences in L2 reading is Zhang’s (2002) investigation. Zhang (2002) used a mixed-methods approach to examine learners’ metacognitive awareness. He found that learners’ confidence, effectiveness, repair strategy, and perception of difficulty influenced their performance. Some researchers have also investigated the role of emotions in relation to the affective dimension of metacognitive experiences in L2 learning (e.g., Choi, 2013; Jiang and Dewaele, 2019; Prior, 2019; Zhang et al., 2022). For example, Jin and Zhang (2021) in examining the dimension of enjoyment in the EFL learning classroom revealed that enjoyment of EFL learning had a positive effect on English achievement.

In the field of L2 writing research, previous studies have endeavored to develop L2 learners’ writing proficiency from the perspective of metacognitive experiences (Kasper, 1997; Lee and Mak, 2018; Dong and Zhan, 2019). Wu’s (2006) research focused specifically on EFL writers’ metacognitive experiences, and not on metacognitive strategy or knowledge, concentrating on the affective experiences. She proposed that EFL writers’ metacognitive experiences were positive and negative feelings. In a recent study, Sun et al. (2021) investigated EFL learners’ after-writing metacognitive experiences by developing a self-report questionnaire. Results of factor analyses identified a four-factor model: metacognitive feelings, metacognitive judgments/estimates, online metacognitive knowledge, and online metacognitive strategies of EFL writing. Unfortunately, they did not map out the dynamics of EFL learners’ metacognitive experiences in writing.

On the whole, it is obvious that learners’ metacognitive experiences have effects on their academic achievements. However, research on metacognitive experiences in L2 writing is still under-researched, particularly for EFL writers who have fewer opportunities to use English for communication. In addition, prior studies on metacognitive experiences in L2 writing have investigated cognitive or affective experiences, but have not established how these affect L2 writing, or specifically examined metacognitive experiences of EFL writing in-depth in the classroom setting. Such research gaps call for empirical evidence of the nature of EFL learners’ metacognitive experiences in writing.

### Assessing metacognitive knowledge and metacognitive strategies in L2 writing

Due to the close connection of metacognitive experience with the other components of metacognition, we also focused on prior research on assessing metacognitive knowledge and metacognitive strategies in L2 writing. Many studies have employed quantitative and qualitative methods for measuring metacognitive factors in L2 writing, such as questionnaires (e.g., Zhang and Qin, 2018) and interviews (e.g., Ruan, 2014). For instance, Ruan (2014) employed small-group interviews to identify the types of Chinese EFL learners’ metacognitive awareness. He claimed that novice EFL writers need to develop strategy awareness of planning, generating, and revising. In addition to using interviews, some researchers sought to employ questionnaires to measure L2 writers’ metacognitive knowledge and metacognitive strategies. Questionnaires are the most frequently employed instruments for assessing metacognition in L2 writing to gather holistic and comprehensive information (e.g., Zhang and Qin, 2018; Teng et al., 2021, 2022).

Researchers have used newly developed questionnaires for assessing metacognitive knowledge and metacognitive strategies in L2 writing (Hwang and Lee, 2017; Karlen, 2017; Zhang and Qin, 2018; Teng, 2020). For example, Teng (2020) developed a self-report instrument with 45 items to measure students’ metacognitive knowledge. The questionnaire required participants to respond to statements on a 7-point Likert scale, from 1 (strongly disagree) to 7 (strongly agree). Three types of metacognitive knowledge were declarative, procedural, and conditional knowledge. The findings of his questionnaire correspond to Schraw’s (1998) categorization of metacognitive knowledge. In a more recent study, Zhao and Liao (2021) developed a 6-point Likert scale with 18 items to investigate L2 writers’ metacognitive strategies use in an authentic writing assessment context. Results revealed that L2 writers’ metacognitive strategies included five types: task interpretation, planning, translating, evaluating and monitoring, and revising.

In sum, many existing studies have identified the nature of metacognitive knowledge and metacognitive strategies in L2 writing. However, there are not many studies that measure metacognitive experiences, a less researched aspect of metacognition, in the context of EFL writing. In addition, researchers usually only measure metacognitive components in L2 writing at one point (i.e., after the writing process). To date, no measures or models have been developed to assess L2 learners’ metacognitive factors before, during, and after the writing process, particularly for EFL writers.

### A hypothesized model of metacognitive experiences in EFL writing

In this study, we propose a new model of EFL writing metacognitive experiences, adapted from Efklides’ (2002a,b) taxonomy of metacognitive experiences in psychological research. We add online task-specific metacognitive strategies to her model because it strengthens the model for the EFL writing context. To capture the nature of EFL writing metacognitive experiences, we based on Efklides’ (2002a,b) and Sun et al.’s (2021) research on metacognitive experiences and further hypothesized that EFL writing metacognitive experiences include four dimensions: (1) *metacognitive feelings*, (2) *metacognitive judgments/estimates*, (3) *online task-specific metacognitive knowledge*, and (4) *online task-specific metacognitive strategies*.

Specifically, we aligned with the first two dimensions of Efklides’ (2002a,b) framework of metacognitive experiences (i.e., metacognitive feelings and metacognitive judgments/estimates). For the third and fourth dimensions of the hypothesized model, we adapted Efklides’ (2002a,b) categorization of *online task-specific knowledge* and added *online task-specific metacognitive strategies*. In our hypothesized model, we renamed *online task-specific knowledge* as *online task-specific metacognitive knowledge* (OTSMK). We use OTSMK because Efklides’ (2002a,b) categorization of online task-specific knowledge is for general cognitive processes, which is inadequate to describe the complexity of EFL writing. Given the interaction and overlapping between metacognitive knowledge, experiences, and strategies in EFL writing, we propose that *online task-specific metacognitive knowledge* consists of *person, task, and strategy knowledge*. Similarly, we added the fourth dimension to the hypothesized model, *online task-specific metacognitive strategies*, which included *planning, monitoring, and evaluating*.

Here, we point out the differences between *online task-specific metacognitive knowledge/strategies* and *metacognitive knowledge/strategies* as these constructs are similar and confusing. First, online task-specific metacognitive knowledge and strategies happen in real time, while metacognitive knowledge and strategies do not occur in real time. Secondly, *online task-specific metacognitive knowledge and strategies* are stored in the short-term memory, while *metacognitive knowledge/strategies* accumulate in the long-term memory. Thirdly, *online task-specific metacognitive knowledge and strategies* are task-specific. The word “task-specific” emphasizes the specific tasks that prompt EFL writing metacognitive-related experiences, whereas metacognitive knowledge and strategies are general terms.

## Methods

Given that Efklides (2002b, 2006b) highlights the dynamic and multifarious nature of metacognitive experiences in the cognitive process, the present study was designed to assess EFL learners’ metacognitive experiences before, during, and after the writing process. Framed with adapted frameworks of metacognitive experiences (Efklides, 2002a,b; Sun et al., 2021) and a hypothesized model, this study aimed to address the following two research questions:

1. What is the taxonomy of EFL learners’ metacognitive experiences in writing?
2. Are the factors of EFL learners’ metacognitive experiences in writing related to their writing performance?

### Participants

The participants were 760 full-time second-year non-English-major undergraduates from seven faculties at a leading national research university in Northeast China. The students were recruited *via* convenience sampling, and their mean age was 19.69 (SD = 0.72). The reason for choosing second-year undergraduates was because they needed to take the College English Test-Band Four (CET-4) following the university requirements. Therefore, these students were motivated to learn how to write in EFL and write more often as examination preparation. All participants had enrolled in an English writing course to prepare for CET-4. The writing course was designed to teach students how to write in EFL and develop their critical thinking ability.

The participants were two independent samples of 340 and 420 who had learned English as a foreign language for an average of 11.53 (SD = 2.11) years. Mandarin Chinese was their first language. Of the sample of 340 participants, 196 were males (57.6%) and 144 (42.4%) were females. They were from the Faculties of Earth Science (*N* = 96, 29.4%), Engineering (*N* = 33, 9.0%), Information Science (*N* = 34, 9.4%), Medical Science (*N* = 69, 20.6%), Science (*N* = 55, 16.1%), and Social Science (*N* = 53, 15.5%). The second sample of participants were 420 undergraduates from four faculties (Engineering *N* = 68, 16.3%; Humanities *N* = 64, 15.2%; Information Science *N* = 145, 34.5%; Science *N* = 143, 34.0%). Among the sample of 420 students, 269 were males (64%) and 151 were females (36%).

### Instruments

#### Self-report questionnaires

In this study, we used three self-report questionnaires to assess EFL learners’ metacognitive experiences before, during, and after the writing process. As there were no existing questionnaires available to measure EFL learners’ metacognitive experiences before and during the writing process, we developed and validated two questionnaires to taxonomize learners’ metacognitive experiences: the Pre-Writing Metacognitive Experiences Questionnaire (PWMEQ) and the During-Writing Metacognitive Experiences Questionnaire (DWMEQ). In addition, we adopted a well-established scale to assess EFL learners’ post-writing metacognitive experiences.

##### The pre-writing metacognitive experiences questionnaire

The PWMEQ was developed following Dörnyei and Taguchi’s (2009) rationale for questionnaire development: item generation, initial piloting, and psychometric evaluation. The process of item generation originated from consulting the existing literature (e.g., Efklides, 2002a,b; Zhang and Qin, 2018) and conducting focus group interviews. We reviewed literature related to researching the construct of metacognitive experiences, the development of self-report instruments to measure metacognition, and the role of metacognitive experiences in EFL writing. Twenty EFL learners’ who were from diverse faculties were invited to describe their learning-to-write experiences. Based on existing literature and learners’ authentic experiences, we generated 38 items related to EFL learners’ pre-writing metacognitive experiences.

Once a preliminary draft of the PWMEQ was completed based on the literature review and interviews, we invited the 20 students to check the clarity and readability. We reworded the questionnaire items with reference to students’ feedback. For example, we revised three double-barreled items. Regarding psychometric evaluation, two experts in the field of L2 writing and educational psychology were invited to scrutinize the items. This procedure resulted in eliminating four items that overlapped or did not match the construct in this study. The revised version of the PWMEQ was a 6-point Likert scale with 34 items, ranging from 1 (strongly disagree) to 6 (strongly agree). The PWMEQ included two sections: students’ demographic information and their pre-writing metacognitive experiences (see Supplementary Appendix A). The questionnaire was developed and administered in English as the participants have a good command of English vocabulary. Translation of questionnaires from English to Chinese might cause slippage.

##### The during-writing metacognitive experiences questionnaire

The DWMEQ was developed to evaluate EFL learners’ during-writing metacognitive experiences (see Supplementary Appendix B). The same procedure was conducted with the DWMEQ as with the PWMEQ: item generation, initial piloting, and psychometric evaluation. On the basis of literature review and focus group interviews, we generated 45 items pertaining to EFL learners’ during-writing metacognitive experiences. After initial piloting and psychometric evaluation, the revised version of the DWMEQ was a 6-point Likert scale with 42 items, rating from 1 (strongly disagree) to 6 (strongly agree).

##### The post-writing metacognitive experiences questionnaire

We used the EFL Learners’ Writing Metacognitive Experiences Questionnaire (EFLLWMEQ), which was a 16-item scale to assess students’ post-writing metacognitive experiences in an EFL learning context (Sun et al., 2021). The questionnaire included four subcategories of post-writing metacognitive experiences: metacognitive feelings (Items 1, 2, 3, and 11), metacognitive estimates (Items 12, 13, 14, 15, and 16), online task-specific metacognitive knowledge (Items 8, 9, and 10), and online task-specific metacognitive strategies (Items 4, 5, 6, and 7). Sun et al. (2021) reported that the EFLLWMEQ was reliable and valid. The internal reliability coefficient values ranged from 0.70 for online task-specific metacognitive knowledge to 0.85 for metacognitive estimates. In our study, the EFLLWMEQ was labeled as the POWMEQ. Supplementary Appendix C lists the items of the POWMEQ.

#### Writing test

A test of argumentative writing was used to examine EFL learners’ writing performance. The writing task (see Supplementary Appendix D) was an argumentative writing task with a given topic adapted from the CET-4. Argumentation is a typical genre used in universities with which students frequently engage, and in the Chinese education system, it is used as the writing subtest of English proficiency tests, such as the CET and the Test for English Majors. This test form is considered effective for evaluating students’ writing performance based on their linguistic competence, critical thinking, and ideas articulation (Hirose, 2003; Teng and Zhang, 2016).

In our study, 420 students were invited to write at least 150 words to address the topic within 30 min in the classroom setting, and then their writing scripts were evaluated by iWrite. iWrite, a web-based automated evaluation tool specially designed for Chinese EFL learners, was used to examine students’ overall writing performance.^1^ The reliability of iWrite and manual marking was 0.90 (Li and Tian, 2018). iWrite generates an overall score for an essay regarding five areas: vocabulary, sentence, organization, content, and mechanics.

### Data collection

At the beginning of our study, 340 students were invited to complete the PWMEQ and the DWMEQ. The two questionnaires, in the traditional paper-and-pencil format, were completed by the participants in the classroom, during an English writing session to ensure authentic context-based information, and to avoid any self-selection bias of online surveys (Dewaele, 2018). It took participants approximately 15–20 min to complete the two questionnaires. Data collected from the PWMEQ and the DWMEQ were used for exploratory factor analysis (EFA).

By the end of the semester, another sample of 420 participants was invited to complete the three questionnaires (i.e., the PWMEQ, DWMEQ, and POWMEQ). On the first day, all participants were asked to complete the PWMEQ. On the second day, participants were required to finish the writing task within 30 min and complete the DWMEQ in the classroom setting. After the writing test, participants were invited to complete the POWMEQ. It took participants around 25–30 min to complete all three questionnaires. In this study, we administered three questionnaires instead of using one questionnaire with three writing stages. This is because students might be fatigued when they need to complete a long questionnaire and a writing task simultaneously. It would take students around 1 h to complete the three questionnaires and the writing task. To this end, we administered three questionnaires separately.

### Data analysis

#### Exploratory factor analysis

Data collected from the PWMEQ and the DWMEQ were analyzed through a series of exploratory factor analyses (EFA) conducted in IBM SPSS Version 26.0. EFA is a useful data reduction technique to detect the latent structure of a relatively large set of variables (Allen et al., 2014; Field, 2018). We employed principal axis factoring (PAF) analysis with direct oblimin rotation to investigate the underlying structures of the two questionnaires (see Tabachnick and Fidell, 2007 for details). Given that the first round of the sample size was over 300, the cut-off value for a significant factor loading in our study was set at 0.32.

#### Confirmatory factor analysis

Confirmatory factor analysis (CFA) was conducted on a sample of 420 participants to cross-validate the measurement model of metacognitive experiences in EFL writing. CFA with Maximum likelihood (ML) estimation was run with IBM SPSS AMOS Version 26.0 to examine the factorial structure underlying the PWMEQ, DWMEQ, and POWMEQ. The model fit was assessed using six indices: the value of the ratio of *χ*^2^ divided by its degree of freedom (*χ*^2^/*df*), comparative fit index (CFI), the goodness of fit index (GFI), Tucker and Lewis coefficient (TLI), root means square’s error of approximation (RMSEA), and standardized root mean square residual (SRMR). According to Hu and Bentler (1999) and Kline (2015), Table 1 shows the threshold of each model fit index indicating an acceptable model fit.

**Table 1:** Goodness of fit indices.

Name of index	*χ*^2^/*df*	RMSEA	GFI	CFI	TLI	SRMR
Cut-off points	<3.0	<0.06	>0.90	>0.90	>0.90	<0.08

#### Pearson product–moment correlation

The Pearson product–moment correlation is a statistical technique to measure the strength of a linear correlation between two variables. In this study, this method was performed to analyze the correlation between EFL learners’ metacognitive experiences in writing and their writing performance. The Pearson product–moment correlation coefficient (*r*) shows the strength of the association (Field, 2018).

## Results

### Factor structures of the three questionnaires

#### Findings from questionnaire on pre-writing metacognitive experiences

Descriptive statistical analysis demonstrated that the mean scores (*M*) of responses to the 34 items of the PWMEQ ranged between 3.05 and 4.82, with standard deviations (SD) ranging from 1.00 to 1.59. The values for skewness and kurtosis were within cut-off values, indicating that all the items in the PWMEQ were normally distributed. Results showed that the KMO value was 0.819 which exceeded the recommended minimum value of 0.60 (Kaiser, 1974), and Bartlett’s Test of Sphericity (Bartlett, 1954) was significant (*p* < 0.001). The KMO measure and Bartlett’s test (KMO = 0.819; *χ*^2^ = 3444.464, *df* = 561, *p* < 0.001) indicated that the correlations between items were sufficient to conduct EFA. Secondly, the inspection of the correlation matrix of questionnaire items indicated that the PWMEQ was suitable for EFA. Thirdly, the initial communalities indicated that the initial communalities of all items were above 0.20 except for Item 9. Thus, Item 9 was removed from further analysis.

We adopted multiple criteria to determine which factors of the PWMEQ could be retained based on existing literature (e.g., Pallant, 2016; Field, 2018). We used Kaiser’s (1960) eigenvalues-greater-than-one (K1) criterion and the scree plot to extract the number of factors. The scree plot, especially the cut-off point, is a reliable technique for factor extraction when the sample size is larger than 200 (Stevens, 1992). As the sample of participants’ size at this stage of the scale development was 310, the scree plot is a reliable approach. Parallel analysis was also adopted to retain factors (Pallant, 2016).

Following the extraction methods, a five-factor scale with 20 items was generated, accounting for 57.09% of the variance, which is considered satisfactory in social science research (Hair et al., 2010). Items with factor loadings >0.32 and items with no cross-loadings were retained. A total of 14 items (Items 2, 4, 5, 7, 8, 9, 14, 15, 20, 21, 22, 23, 24, and 25) were excluded from further analysis. The communalities of all extracted variables were higher than 0.30, which revealed that the extracted factors accounted for a sufficient proportion of the variables’ variance (Pallant, 2016). Table 2 presents the results of EFA and the reliability of the PWMEQ.

**Table 2:** Results of EFA and the reliability of the PWMEQ.

Factor (Theme)	Item	Factor loading				
		1	2	3	4	*α*
PWMEQ-Factor 1	Item27	0.836				0.832
	Item28	0.812				
	Item29	0.762				
	Item26	0.651				
	Item30	0.457				
	Item16	0.339				
PWMEQ-Factor 2	Item33		0.733			0.684
	Item31		0.693			
	Item32		0.528			
	Item13		0.408			
PWMEQ-Factor 3	Item12			0.547		0.607
	Item6			0.535		
	Item10			0.500		
	Item34			0.479		
PWMEQ-Factor 4	Item19				−0.761	0.693
	Item18				−0.645	
	Item17				−0.530	

Items with a factor loading of 0.32 or greater are included; *α*, Cronbach’s alpha; PWMEQ, pre-writing metacognitive experiences questionnaire.

CFA was employed to check the construct validity of the PWMEQ. All assumptions for conducting CFA were examined, and no multivariate outliers were detected through the computation of Mahalanobis distance (Tabachnick and Fidell, 2007; Kline, 2015). Initial CFA results, based on goodness-of-fit indices, indicated an overall inadequate model fit (*χ*^2^ = 263.256; *df* = 97; *p* < 0.001; *χ*^2^/*df* = 2.714; TLI = 0.880; CFI = 0.903; RMSEA = 0.072; SRMR = 0.0666). In reviewing parameters of covariance, we noticed that covariance between Item 29 and 30 (e 29 and e 30) was large. Despite the small difference, Item 29 and Item 30 were both relevant to feelings of difficulty in writing content; error correlations between Item 29 and Item 30 were included.

**Figure 1:**
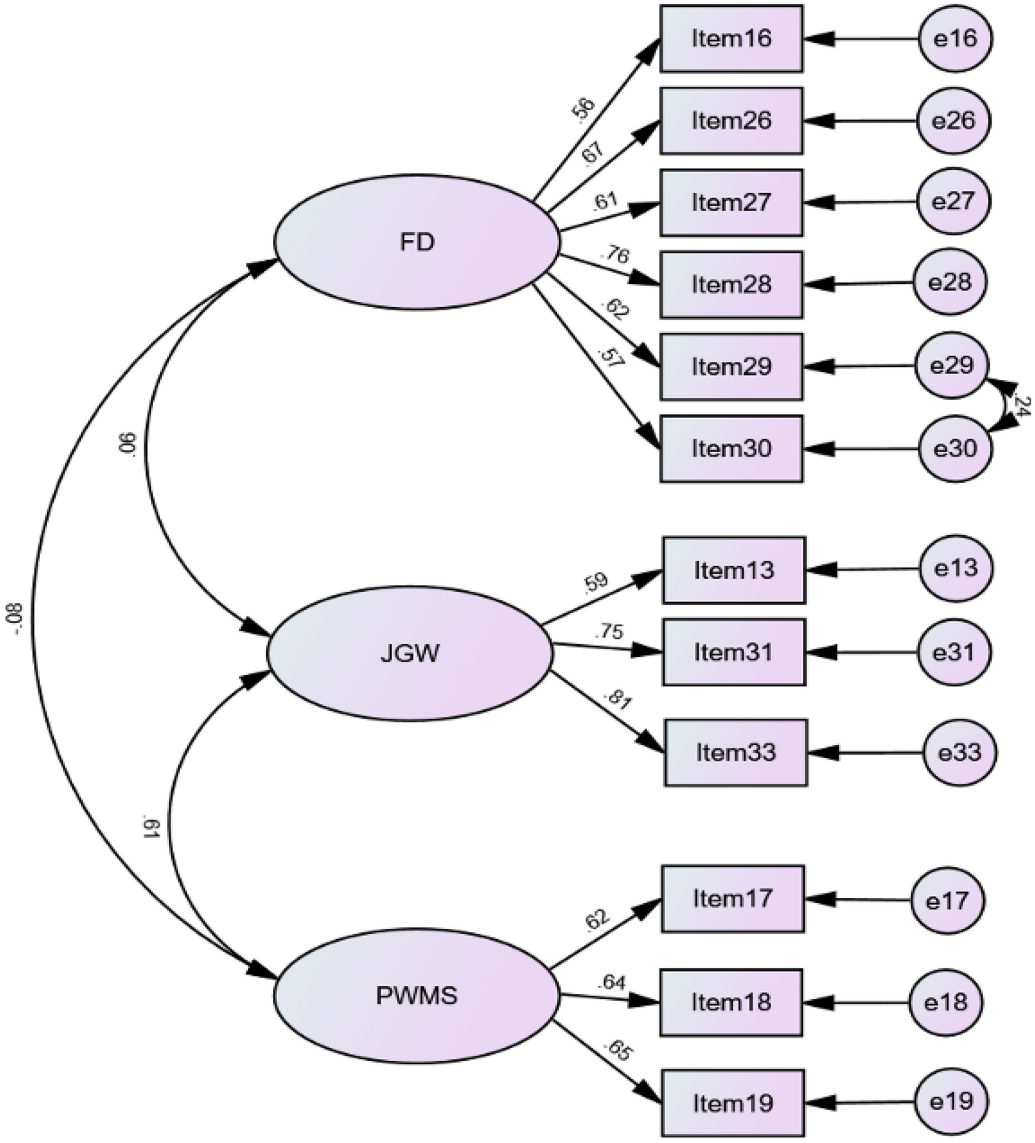
A three-factor correlated model of pre-writing metacognitive experiences. FD, feeling of difficulty; JGW, judgments of good writing; PWMS, pre-writing metacognitive strategies.

In reference to the convergent validity of the PWMEQ, the recommended value of standardized regression weights (i.e., factor loading) adopted in this research was 0.50 (Raykov and Marcoulides, 2008; Awang, 2012). Due to the minimum item factor loading requirement for each factor, Items 6, 10, 12, and 34 were eliminated. Item 32 was also removed as the factor loading (0.32) was smaller than the cut-off point of 0.50. The CFA results of 12-item scale fitted the data well with *χ*^2^ = 145.042; *df* = 50; *p* < 0.001; *χ*^2^/*df* = 2.901; TLI = 0.911; CFI = 0.933; RMSEA = 0.066; SRMR = 0.0583. Figure 1 presents results for the three-factor correlated model of the PWMEQ. All 12-item parameter estimates were significant at *p* < 0.001, and standardized estimates loading were generally higher than the benchmark 0.50, showing a large effect size (Raykov and Marcoulides, 2008).

The results of CFA cross-validated the structure of the PWMEQ. Three factors were generated: feeling of difficulty, judgments of good writing, and pre-writing metacognitive strategies.

##### Feeling of difficulty

Factor One was feeling of difficulty, which refers to EFL learners’ feeling of difficulty in learning to write. The first factor comprised six items (Items 16, 26, 27, 28, 29, and 30). Of these six items, students’ feeling of difficulty was related to grammar use (Item 27), content (Items 28, 29, and 30), and vocabulary use (Items 26 and 16). Referring to Efklides’ (2002a,b) taxonomy of metacognitive experiences, the first factor, affective experiences, is a subcategory of metacognitive feelings. Before writing, students drew on previous writing experiences and experienced negative metacognitive feelings. Findings from extant studies have illustrated that positive emotions can assist the process of language teaching and learning (Dewaele and Alfawzan, 2018; Richards, 2020). However, learners’ feelings of difficulty impede the fluency of problem-solving processes (Efklides, 2006a; Efklides and Vlachopoulos, 2012).

##### Judgments of good writing

The second factor was labeled as judgments of good writing, involving three items: EFL learners’ judgments of good writing from the perspectives of content (Item 33), vocabulary use (Item 31), and grammar correctness (Item 13). Students had been taught linguistic knowledge in the EFL learning context. In the Chinese EFL learning context, affected by Confucian values, teachers are recognized as authorities and experts to guide their students’ learning process in the classroom setting (Zhang, 2002; Hu, 2014). Teachers have taught students vocabulary use and grammar correctness of EFL writing. As such, Chinese EFL learners pay more attention to linguistic knowledge in writing performance, compared to what they give to the organization. The finding of our research aligns with Zhang’s (2010) argument that global aspects such as organization seemed to be missing for EFL learners.

##### Pre-writing metacognitive strategies

The third dimension of the PWMEQ including three items (Items 17, 18, and 19) was named as pre-writing metacognitive strategies, referring to the strategies students intended to use in their writing process premised on their previous writing experiences. Before composing, students retrieved metacognitive strategies from their previous experiences and tried to deploy those strategies in new writing tasks. Some previous studies have shown that learners’ prior experiences map onto the new situation and enable learners to prepare for new tasks (Taylor and Drury, 2004). This result reveals that students’ prior experiences could provide insight into developing EFL writing instruction. Furthermore, the finding of this dimension also showed EFL writers’ metacognitive awareness of strategy use, which is consistent with Zhang and Qin’s (2018) argument that EFL learners orchestrate discourse planning and local lexical planning.

#### Findings from questionnaire on during-writing metacognitive experiences

Descriptive statistical analyses demonstrated that the mean scores of 42 items ranged from 2.90 (SD = 0.93) to 4.88 (SD = 1.48). The values of skewness and kurtosis were within the cut-off points range of |3.0| and |8.0| respectively (Kline, 2015), indicating the data were normally distributed. Moreover, assumptions of normality, linearity, and homogeneity of the sample were also examined, and no outliers were found.

The KMO value was 0.908, exceeding the recommended value of 0.6 (Kaiser, 1974) and Bartlett’s Test of Sphericity (Bartlett, 1954) reached statistical significance (*χ*^2^ = 5324.213, *df* = 861, *p* < 0.001). The initial communalities of all items of the DWMEQ ranged from 0.264 to 0.670, which was higher than the benchmark value of 0.20 (Allen et al., 2014). Cronbach’s alpha coefficients for the five factors ranged from 0.677 for DWMEQ-Factor 3 to 0.874 for DWMEQ-Factor1. Table 3 shows the EFA results of each subscale and internal reliabilities of the DWMEQ.

**Table 3:** Results of EFA and the reliability of the DWMEQ.

Factor (Theme)	Item	Factor loading					
		1	2	3	4	5	*α*
DWMEQ-Factor 1	Item 12	0.705					0.874
	Item 41	0.671					
	Item 18	0.586					
	Item 19	0.571					
	Item 11	0.494					
	Item 38	0.493					
	Item 13	0.394					
DWMEQ-Factor 2	Item 3		0.792				0.691
	Item 1		0.689				
	Item 2		0.580				
	Item 40		0.410				
DWMEQ-Factor 3	Item 25			0.743			0.677
	Item 26			0.581			
	Item 27			0.490			
	Item 21			0.442			
DWMEQ-Factor 4	Item 5				0.786		0.695
	Item 4				0.708		
	Item 7				0.441		
DWMEQ-Factor 5	Item 14					−0.880	0.693
	Item 15					−0.615	
	Item 31					−0.404	

Items with a factor loading of 0.32 or greater are included; *α*, Cronbach’s alpha; DWMEQ, during-writing metacognitive experiences.

**Figure 2:**
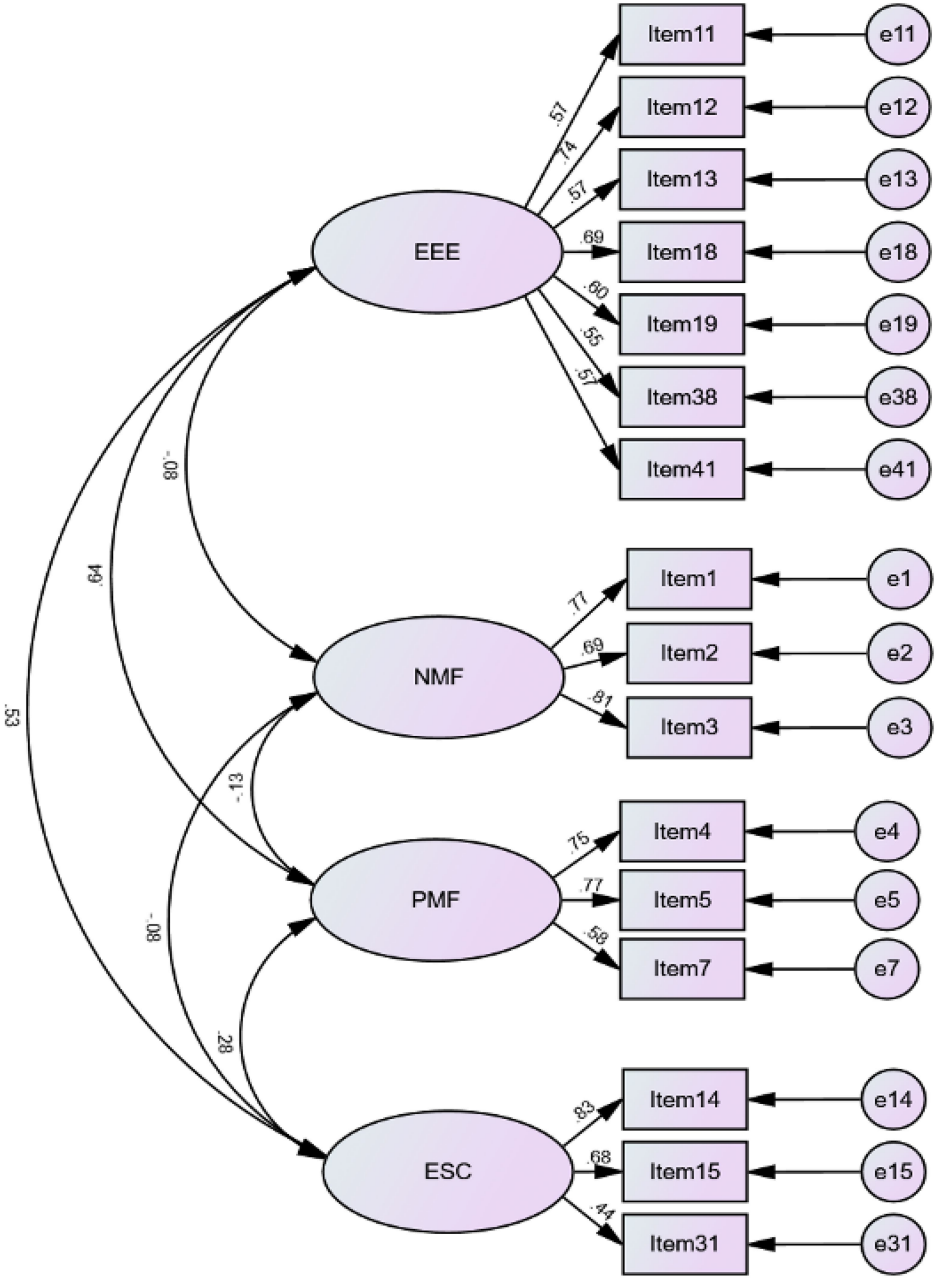
A four-factor correlated model of during-writing metacognitive experiences. EEE, estimate of effort expenditure; NMF, negative metacognitive feelings; PMF, positive metacognitive feelings; ESC, estimate of solution correctness.

CFA was adopted to examine the five-factor model that was generated as a hypothesized model from EFA. The CFA results (*χ*^2^ = 416.990; *df* = 175; *p* < 0.001; *χ*^2^/*df* = 2.383; TLI = 0.877; CFI = 0.898; RMSEA = 0.056; SRMR = 0.0667) for the five-factor model with the 21 items suggested that the model fit was not fully satisfactory. With reference to standardized regression weights, the values of factor loading for Factor 3 of the DWMEQ including Items 25, 26, 27, and 21 were not greater than the benchmark values of 0.50 of factor loadings, so those four items were deleted from the DWMEQ. The modification indices showed that better model fit could be achieved with the removal of Item 40 as the factor loading was much smaller than the cut-off point of 0.50. The final CFA results of four-factor correlated model of the DWMEQ revealed an acceptable model fit with *χ*^2^ = 212.439; *df* = 98; *p* < 0.001; *χ*^2^/*df* = 2.168; TLI = 0.931; CFI = 0.943; RMSEA = 0.052; SRMR = 0.0474 (Figure 2).

The results of CFA provided substantial evidence for the factorial structure of the DWMEQ, entailing estimate of effort expenditure, negative metacognitive feelings, positive metacognitive feelings, and estimate of solution correctness.

##### Estimate of effort expenditure

The first factor of the DWMEQ was labeled as estimate of effort expenditure and was composed of seven items (Items 11, 12, 13, 18, 19, 38, and 41). The factor denoted students’ effort allocation concerning lexical and syntactic use, grammar correctness, organization, and task knowledge. This result lends support to some existing studies that L2 learners have clear metacognitive awareness about their learning process, including with their L2 writing (Ruan, 2014; Amini et al., 2020). The results of our study enrich the framework of metacognitive experiences in EFL writing as described by Efklides’ (2002a,b) taxonomy of metacognitive experiences.

##### Negative metacognitive feelings

The second factor of during-writing metacognitive experiences was defined as negative metacognitive feelings comprising three items (Items 1, 2, and 3). This factor depicts EFL writers’ disengagement feelings as linked to anxiety, chaos, and aversion during the writing process. Given the complexity of writing, these negative feelings may impede students’ successful writing performance. Previous studies have found that learners’ negative emotions have an adverse impact on their writing performance (e.g., Zabihi, 2018; Jin and Zhang, 2021). This finding may provide insights into EFL writing instruction that is influenced by negative metacognitive feelings.

##### Positive metacognitive feelings

The third factor of the DWMEQ was positive metacognitive feelings comprising three items (Items 4, 5, and 7). This finding aligns with Efklides’ (2002a,b) theoretical framework of metacognitive experiences, which provides insights that are applicable to understanding the kinds of feelings EFL writers produce during the writing process. What is noteworthy here is that EFL learners experienced feelings of confidence and familiarity as well. The finding is consistent with previous research on metacognitive experiences in problem-solving (e.g., Efklides, 2006a,b, 2009). Positive affects provide resources for effort exertion (Efklides et al., 2017). In a similar vein, positive metacognitive feelings play a key role in writing performance.

##### Estimate of solution correctness

Factor four of during-writing metacognitive experiences, estimate of solution correctness, included three items (Items 14, 15, and 31), which refers to students’ estimates of their writing quality in the process of composing. This result indicates that, while writing, students scrutinize and monitor their use of lexicon, syntax, and grammar use in writing, paying attention to linguistic accuracy not only after the writing process but also during the process of composing. This finding showed that EFL learners had the ability to judge their writing performance (i.e., the quality of their answers) in EFL writing. As previous research has demonstrated, our finding indicates the benefits of self-reflection in improving learning outcomes (Akama and Yamauchi, 2004; Negretti, 2017).

#### Findings from questionnaire on post-writing metacognitive experiences

**Figure 3:**
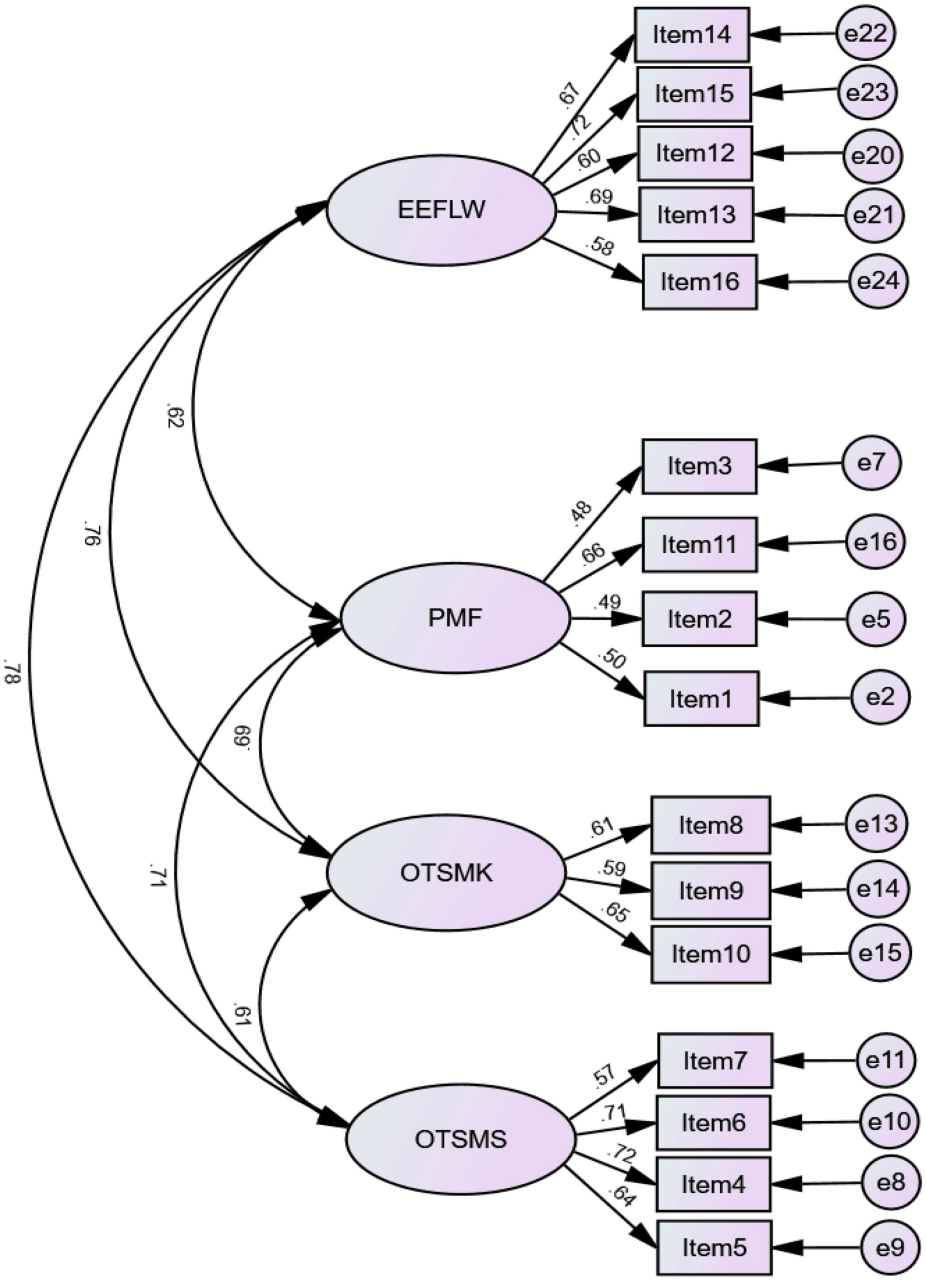
A four-factor correlated model of post-writing metacognitive experiences. EEFLW, estimates of EFL writing; PMF, positive metacognitive feelings; OTSMK, online task-specific metacognitive knowledge; OTSMS, online task-specific metacognitive strategies.

In line with Sun et al. (2021), a four-factor model of post-writing metacognitive experiences was examined. The model adequately fits the data (*χ*^2^ = 239.359; *df* = 98; *p* < 0.001; *χ*^2^/*df* = 2.442; TLI = 0.908; CFI = 0.925; RMSEA = 0.059; SRMR = 0.0530), including metacognitive estimates, positive metacognitive feelings, online task-specific metacognitive knowledge, and online task-specific metacognitive strategies. Figure 3 shows the standardized regression weights of the measurement model.

#### Relationships between EFL learners’ metacognitive experiences and their writing performance

Correlations between the 11 factors of EFL writing metacognitive experiences and writing performance were examined. The Pearson correlation coefficients in Table 4 suggest that EFL learners’ writing performance was weakly correlated with estimate of effort expenditure (*r* = 0.045), positive metacognitive experiences (during-writing, *r* = 0.096), estimates of EFL writing (*r* = 0.050), and online task-specific metacognitive knowledge (*r* = 0.203). The significant correlations confirmed the predictive validity of the questionnaires.

**Table 4:** Pearson’s correlation coefficients on the 11 factors of metacognitive experiences and writing performance.

		FD	JGW	PWMS	EEE	NMF	PMF (during writing)	ESC	EEFLW	PMF (after writing)	OTSMK	OTSMS
Writing performance	Pearson’s correlation	0.016	−0.015	0.058	0.045^*^	0.076	0.096^**^	0.045	0.050^*^	0.093	0.203^**^	0.118

FD, feeling of difficulty; JGW, judgments of good writing; PWMS, pre-writing metacognitive strategies; EEE, estimate of effort expenditure; NMF, negative metacognitive feelings; PMF, positive metacognitive feelings (during writing); ESC, estimate of solution correctness; EEFLW, estimates of EFL writing; PMF, positive metacognitive feelings (after writing); OTSMK, online task-specific metacognitive knowledge; OTSMS, online task-specific metacognitive strategies.

* *p* < 0.05 (2-tailed),

** *p* < 0.01 (2-tailed).

## Discussion

**Figure 4:**
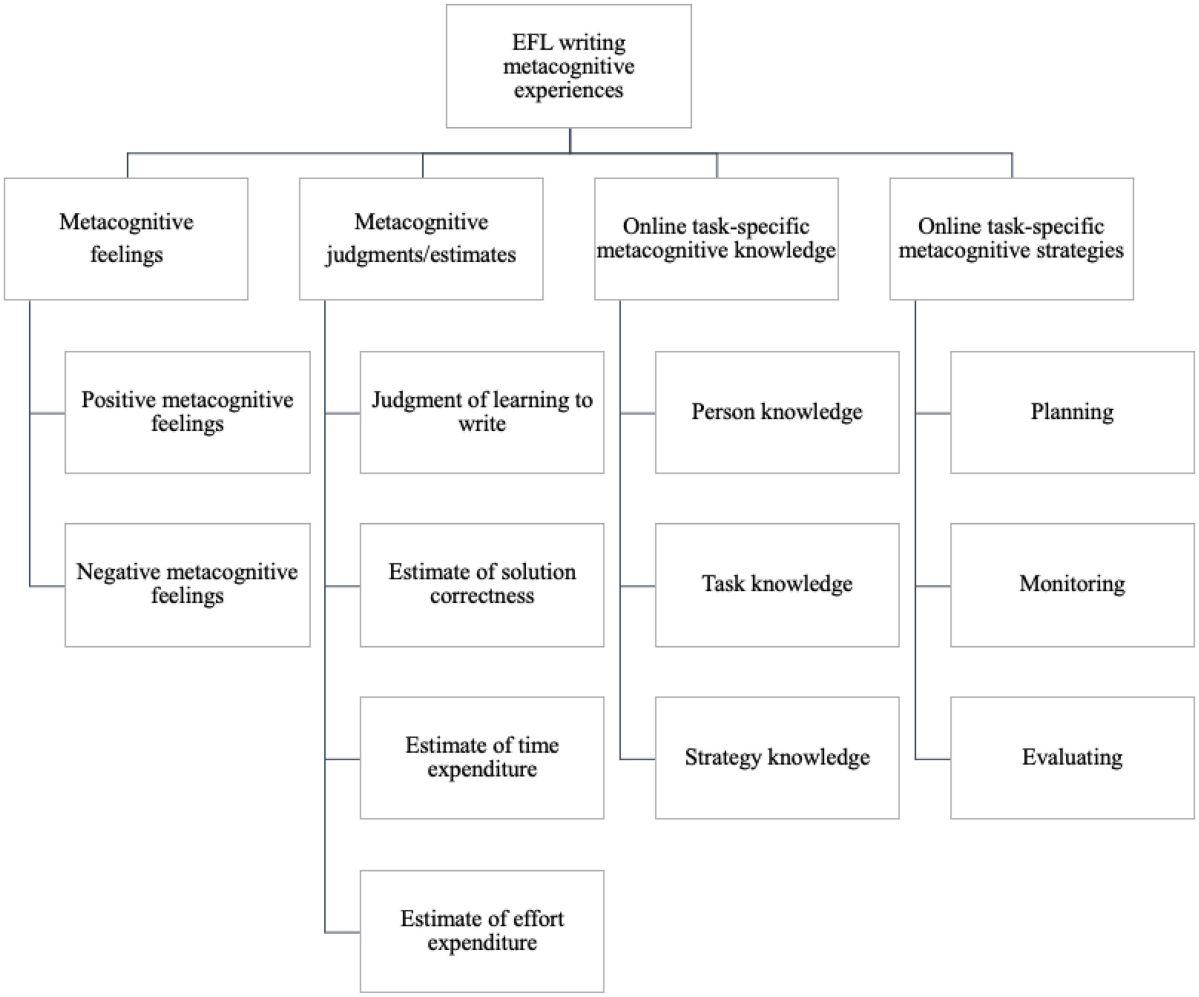
A model of EFL learners’ metacognitive experiences in writing.

This study was designed to assess EFL learners’ metacognitive experiences before, during, and after the writing process. Results of CFA indicated that EFL writing metacognitive experiences could be categorized into 11 subcategories. On the basis of the empirical evidence, we propose a model to conceptualize EFL writers’ metacognitive experiences including metacognitive feelings, metacognitive judgments/estimates, online task-specific metacognitive knowledge, and online task-specific metacognitive strategies (Figure 4).

### Metacognitive feelings

EFL writers experienced both positive and negative metacognitive feelings, as expected of the affective dimension of metacognitive experiences. This finding adds new dimensions to metacognition studies by gathering reliable evidence of EFL writers’ affective experiences. Our study indicates that metacognitive experiences encompass both affective and cognitive experiences, corroborating Flavell’s (1979) and Efklides’ (2002a,b) theoretical models. These empirical findings support Efklides et al.’s (2017) proposition that metacognition and affect overlap rather than exist in two distinct strands. Furthermore, findings revealed that metacognitive feelings happened before, during, and after the writing process. These empirical results of our study, in line with Efklides’ (2002a,b, 2006a) theoretical argument that metacognitive experiences are dynamic, enrich understandings of EFL learners’ metacognitive feelings in learning to write.

What is noteworthy in our study is that data collected from the three self-report questionnaires provide a comprehensive picture of metacognitive feelings. Results of factor analyses showed EFL writers experienced feelings of difficulty, satisfaction, familiarity, and confidence, reflecting Efklides’ (2002a,b, 2006b, 2008) theoretical claims and adding to her models. The findings of metacognitive feelings in our study lend empirical support to investigations into the role of affective experiences in the learning process, in which metacognitive feelings were found to affect learners’ academic performance (Zhang, 2002; Wu, 2006; Efklides and Vlachopoulos, 2012; Dong and Zhan, 2019; Sun and Zhang, 2022). Similar to some prior studies on L2 learning (Zhang and Zhang, 2013; Jiang and Dewaele, 2019; Jin and Zhang, 2021), the findings of our study reveal that these metacognitive feelings may support learning to write in EFL. Dong and Zhan (2019) also found EFL learners’ positive metacognitive feelings exerted a positive effect on their writing scores, suggesting there is a need for instructors and EFL learners to develop positive metacognitive feelings about writing. For example, instructors’ classroom talk could focus on positive affective language, such as “notice how you are building your writing skills every time you take on another writing task.”

Whereas such talk is likely to be helpful in most classrooms, we suggest that it is particularly helpful in Chinese EFL writing pedagogy. Students’ metacognitive feelings are normally ignored by researchers and instructors in the Confucian culture learning contexts because teachers are considered the authorities (Kennedy, 2002), and their praise or explicit acknowledgment of development is likely to have greater impacts. In this regard, the influence of the teachers’ role in EFL writing, such as their feedback and teaching approaches, are likely to affect writers’ affective experiences. Findings of affective experiences in our study indicate that EFL writing instructors’ role in establishing positive emotions is crucial in learning to write.

### Metacognitive judgments/estimates

The second dimension, metacognitive judgments/estimates, aligning with Efklides’ (2002a,b) framework from the perspective of cognitive experiences, consisted of (1) judgments of learning to write in EFL; (2) estimate of solution correctness; (3) estimate of time expenditure, and (4) estimate of effort expenditure. Similar to metacognitive feelings, metacognitive judgments/estimates occurred before, during, and after the writing process. There were relationships among EFL writers’ judgments of good writing (pre-writing), estimate of effort expenditure (during-writing), and estimates of EFL writing (post-writing). EFL writers who can accurately judge good EFL writing are likely to have accurate estimates of their effort expenditure to complete writing tasks; they are likely, also, and to accurately calibrate their writing performance after finishing writing tasks.

The first subcategory of metacognitive judgments/estimates was judgments about learning to write in EFL. Quantitative results of EFL writers’ responses to the PWMEQ indicated their reported judgments of what good writing was, including vocabulary use, sentence structures, grammar correctness, and logical flow. In addition, participants in reporting their estimate of solution correctness during and after the writing process focused predominantly focusing on their linguistic performance, corroborating from a theoretical perspective, Efklides’ (2002a,b) taxonomy of metacognitive experiences. Participants self-judged their writing processes by checking linguistic accuracy and logical content. After completing writing tasks, they reported comprehensive estimates of their EFL writing performance, including vocabulary use, sentence structure, grammar correctness, logic of writing content, and their time expenditure, as noted in previous studies on L2 writing (Wong, 1999; Weigle, 2005; Zhang et al., 2014). Writing has been recognized as a hybrid of cognitive and metacognitive activity (Flower and Hayes, 1981), with metacognition generally including both self-appraisal and self-management. Participants in our study tended to have an awareness of self-evaluation to improve their writing performance. Hayes’ (2012) cognitive model of writing emphasizes the crucial role of metacognition in writing; the findings of our study likewise reveal the importance of developing EFL writers’ awareness of metacognitive estimates.

EFL writers’ estimate of time expenditure was apparent in the examination situation. Consistent with Efklides’ (2002a,b) framework of metacognitive experiences, EFL writers paid attention to the time needed for completing a writing task, due possibly to the prevalence of an examination culture in the Chinese EFL context. EFL writers in this study were likely to be crucially aware of time expenditure, ensuring that they apportioned their time appropriately, as they would take CET. Participants also demonstrated estimating their expenditure of effort (i.e., effort allocation) with reference to personal knowledge, vocabulary use, organization, and task requirements. Drawing on their previous experiences, for example, what they had learned and used in learning to write when composing their writing. The findings of our study support the results of previous studies which found that L2 writers, who were aware of what information could be used to accomplish writing tasks, were more likely to perform successfully in completing the tasks (Anderson, 2003; Negretti, 2012; Ruan, 2014).

### Online task-specific metacognitive knowledge

The third dimension, online task-specific metacognitive knowledge related to person, task, and strategy knowledge, is retrieved specifically from working memory. The findings of our study also demonstrated that EFL learners’ online task-specific metacognitive knowledge occurred before, during, and after the writing process. This dimension resonates with Efklides’ (2002a,b) framework of metacognitive experiences regarding online task-related knowledge. It is noteworthy that participants also reported their person and strategy knowledge in EFL writing. These results reveal evidence in our study that is congruent with existing studies on general metacognitive knowledge in L2 writing (Victori, 1999; Negretti and Kuteeva, 2011; Negretti and McGrath, 2018; Teng, 2020), online task-specific metacognitive knowledge playing a pivotal role in EFL writing.

### Online task-specific metacognitive strategies

Whereas Efklides (2002a,b) proposed three subcategories of metacognitive experiences in the cognitive process, a novel contribution of our study is that the quantitative results enable us to propose a fourth dimension of EFL writing metacognitive experiences, i.e., online task-specific metacognitive strategies. Demarcation of metacognitive components can be arbitrary because, in the process of writing, metacognitive experiences, metacognitive knowledge, and metacognitive strategies are intertwined (Lee and Mak, 2018). However, Efklides (2002a,b) did not take online task-specific metacognitive strategies into consideration even though these strategies were crucial, particularly in the process of EFL writing. Therefore, in the model of EFL writing metacognitive experiences, the findings enabled us to propose online task-specific metacognitive strategies.

In our study, participants reported three subcategories of online task-specific metacognitive strategies: planning, monitoring, and evaluating, which have also been reported in previous research on metacognitive strategies in L2 learning (Bai et al., 2014; De Silva and Graham, 2015; Amini et al., 2020; Zhao and Liao, 2021). EFL writers in this study tended to plan before writing and monitor their writing process and evaluate their language use after finishing writing tasks; it is noted that online task-specific metacognitive strategies can also be conscious processes. They did not, however, tend to self-evaluate their ideas or thoughts much. These participants reported they paid attention to error correction in examinations.

Taken together, the results of EFL writers’ perceived metacognitive experiences not only provide empirical evidence for Efklides’ (2002a,b) framework of metacognitive experiences but also enrich the understanding of EFL writing metacognitive experiences. The results of our study captured the way that EFL learners’ metacognitive experiences happened before, during, and after the writing process.

## Conclusion

Conceptualized in the frameworks of metacognitive experiences, this study used a quantitative approach to assess EFL learners’ metacognitive experiences before, during, and after the writing process. The results provided empirical evidence on the taxonomy of EFL learners’ metacognitive experiences: metacognitive feelings, metacognitive judgments/estimates, online task-specific metacognitive knowledge, and online task-specific metacognitive knowledge. Our study contributes to a better understanding of EFL learners’ metacognitive experiences before, during, and after the writing process.

The findings might have theoretical, methodological, and pedagogical implications. Theoretically, this study contributes to advancing the field of EFL writing research by applying the framework of metacognitive experiences from educational psychology to the study of EFL writing. We propose a four-dimension EFL writing metacognitive experiences model. From a methodological perspective, the two newly developed questionnaires (i.e., the PWMEQ and the DWMEQ) were designed and validated, providing a new method for assessing EFL learners’ metacognitive experiences before and during the writing process.

The model of EFL writing metacognitive experiences proposed in this study provides pedagogical implications for EFL instructors and syllabus designers for improving learners’ writing performance and developing self-regulatory competence. First, the two newly developed questionnaires contributed by our study might be adopted as self-assessment tools to measure the richness of EFL writers’ metacognitive experiences. Based on the scores calculated from the three questionnaires, students can adjust and reconstruct their metacognitive experiences to expedite their learning-to-write processes. As part of improving teaching practice, the three questionnaires could also be used to diagnose EFL writers’ metacognitive experiences before the intervention, thus providing insights into how EFL writing could be taught to students. Inviting students to undertake this self-assessment would alert them to metacognitive experiences as another factor influencing their learning of EFL writing. Secondly, the findings of this research reveal the potential of considering EFL learners’ psychological dimensions such as metacognitive feelings in curriculum design and instructional practices.

Although rich data have been collected in this study, some limitations still remain. First, second-year undergraduates were recruited from only one university in mainland China. As they were preparing for CET-4, these participants were strongly motivated to learn to write in EFL. Thus, the findings from this research may not be broadly generalized and applicable in L2 and other EFL writing contexts where there is less motivation. It is recommended that future research is undertaken in different learning contexts to assess learners’ metacognitive experiences in writing. In addition, we only adopted questionnaires to assess learners’ metacognitive experiences in writing. As is understood, self-report instruments cannot capture the dynamic nature of metacognitive components of the complex L2 writing process. Future research is needed to employ multiple methods. For example, researchers can employ interviews and think-aloud protocols.

## Author’s note

QS has recently completed her PhD in Education (Applied Linguistics and TESOL) at the Faculty of Education and Social Work, the University of Auckland, Auckland, New Zealand. She has just started her first academic job as a lecturer/assistant professor at the School of Foreign Language Education, Jilin University, China. Her research interests include second language acquisition, especially L2 written language acquisition, ESL/EFL writing, and metacognition in language learning. Her publications have appeared in *Language Teaching Research* (Sage), *Current Psychology* (Springer Nature), and *Frontiers in Psychology* (Frontiers media). LZ, PhD, is a Professor of Linguistics-in-Education and Associate Dean for the Faculty of Education and Social Work, The University of Auckland, New Zealand. His major interests and 100-plus publications are on learner metacognition, language-teacher education, and L2 reading-writing development. He is Co-Chief-Editor for *System* (Elsevier) and an associate editor for *Frontiers in Psychology,* serving as an editorial board member for journals such as *Applied Linguistics Review* (de Gryuter), *Australian Review of Applied Linguistics* (Benjamins), *Chinese Journal of Applied Linguistics* (de Gruyter)*, Journal of Second Language Writing* (Elsevier), *Metacognition and Learning* (Springer Nature), *Journal of Second Language Studies* (Benjamins), *Asian-Pacific Journal of Second and Foreign Language Education* (Springer Nature), *Asian Journal of English Language Teaching* (CUHK), *English Teaching and Learning* (Springer Nature), and *RELC Journal* (Sage). He was honored by the TESOL International Association (United States) in 2016 with the award of “50 at 50,” acknowledging “50 Outstanding Leaders” and was officially installed as a newly elected member of the Board of Directors of the Association in 2017.

## Data availability statement

The original contributions presented in the study are included in the article/Supplementary material, further inquiries can be directed to the corresponding author.

## Ethics statement

The studies involving human participants were reviewed and approved by The Human Ethics Committee of the University of Auckland, New Zealand. The patients/participants provided their written informed consent to participate in this study.

## Author contributions

All authors listed have made a substantial, direct, and intellectual contribution to the work and approved it for publication.

## Funding

This study was supported by a New Zealand China Doctoral Research Scholarship (NZCDRS) for the first author to complete her Ph.D. study at the University of Auckland, New Zealand.

## Conflict of interest

The authors declare that the research was conducted in the absence of any commercial or financial relationships that could be construed as a potential conflict of interest.

## Publisher’s note

All claims expressed in this article are solely those of the authors and do not necessarily represent those of their affiliated organizations, or those of the publisher, the editors and the reviewers. Any product that may be evaluated in this article, or claim that may be made by its manufacturer, is not guaranteed or endorsed by the publisher.

## Footnotes

^1^ http://iwrite.unipus.cn/
